# Physical activity in the Families in Transformation (FIT) weight management program for children

**DOI:** 10.15171/hpp.2018.32

**Published:** 2018-07-07

**Authors:** Kathy B. Knight, Sydney A. Devers, Meagan Maloney, Anne K. Bomba, Heather Walker, Kathy Tucker, Scott S. Knight

**Affiliations:** ^1^Department of Nutrition and Hospitality Management, University of Mississippi, University, MS, USA; ^2^Coordinator of Outreach and Innovation, Health Works! North Mississippi, North Mississippi Health Services, Tupelo, MS, USA; ^3^Director of University of Mississippi Field Station, University of Mississippi, University, MS, USA

**Keywords:** Physical activity, Fitness, Pediatrics, Nutrition education

## Abstract

**Background:** The purpose was to determine if an 8-week nutrition education and exercise program for families could influence health and fitness parameters, and retention of nutrition knowledge.

**Methods: ** Eighteen children (mean age: 10.52 ± 1.26 year; 50% boys, 50% girls; 56% white, 25% black, 19% multiracial) participated in the Families in Transformation (FIT) program. Preand post-study anthropocentric, blood pressure, fitness, and nutrition knowledge data was collected.

**Results: ** Diastolic blood pressure decreased for the total group (66.63 ± 8.81 to 63.75 ± 11.81mm Hg). Significant (P < 0.05) increases were seen for the group for push-ups (14.31 ± 7.62 to 19.63 ± 6.62) and chair squats (30.50 ± 10.21 to 34.44 ± 7.39). The reinforcing physical activity group performed significantly better on nutrition knowledge quizzes.

**Conclusion:** Although, body mass index (BMI) z-scores did not change, there was a decrease in diastolic blood pressure, increase in fitness parameters, and increased retention of nutrition knowledge.

## Introduction

According to the Centers for Disease Control and Prevention (CDC),^1,2^ 17.7% of American children ages 6-11 are obese. The principal cause of childhood obesity, too many calories consumed and inadequate calories burned, is a multidimensional problem affected by factors such as genetics, metabolism, and nutrition and physical activity behaviors.^2^ Furthermore, children often remain obese into adulthood, resulting in increased morbidity and mortality and increase health care costs.^2,3^

As can be imagined, health professionals – both practitioners and educators – are concerned and looking for options to combat this epidemic. In their recommendations for the Pediatric Weight Management Nutrition Practice, the Academy of Nutrition and Dietetics recommended multidisciplinary interventions that included physical activity, diet and nutrition counseling, and patient participation.^4^ They also suggested that the most successful interventions include both nutrition and physical activity as physical activity alone has not been as effective with children. While sustained programs that deliver successful, long-term results are needed to combat this epidemic, effective short-term programs with a focus on physical activity and nutrition education may be a useful referral resource for health care professionals with obese or at-risk patients in need of prevention/treatment resources.^3^ While short-term obesity prevention programs may not always result in a reduction in body weight; other positive effects may be seen including greater program compliance,^4^ a reduction in adiposity, improvement in cardiovascular and diabetes risk factors, and an increase in healthy behaviors and physical fitness.^5-10^

One factor that may reflect the efficacy of an obesity prevention program is the retention of nutritional knowledge given to children, with the most successful programs including behavior modeling and participatory activities to increase information retention.^11^ It is suggested that children better grasp new concepts through hands-on-learning experiences as opposed to simply hearing information.^12^ According to the United States Department of Agriculture (USDA), nutrition education can be more effective when combined with physical education,^13^ and the USDA *ChooseMyPlate.gov* website highlights the importance of both nutrition and physical education, offering interactive nutrition quizzes, nutrition activities, and athletic games that reinforce nutrition education.^14^

The purpose of this study was to measure the effectiveness of physical activity in a short-term nutrition education and physical activity program for children and their families, and to determine if physical activity can reinforce the nutrition education that would be offered in such a program.

## Materials and Methods

Researchers from the Department of Nutrition and Hospitality Management at the University of Mississippi (UM NHM) conducted an evaluation of a childhood obesity prevention program entitled *Families In Transformation (FIT)* provided by the *HealthWorks!* health education and fitness center in Tupelo, Mississippi*.* The program had a $25 participation fee, but upon completion of the program, the participation fee was returned and each family received a $100 grocery store gift card from *Healthworks!*

### Participants

In the fall of 2016, the *HealthWorks!* staff recruited children and their parents from schools and pediatric and family medicine clinics in and near Tupelo. All children who were referred by their school nurse or area physician were allowed to join the program and agreed to be participants in this study. The 18 participants, which represented 100% of all the children in the program were from 8 to 11 years of age (Mean age was 10.52 ± 1.26.), and as seen in Table 1, both genders were almost equally represented and there was some ethnic diversity. Some of the children were overweight as identified by body mass index (BMI) z-scores (n = 6 females and 2 males). Some were referred to the program because of obesity risk (overweight) and high blood pressure (n = 3 females and 4 males), and/or poor nutrition and/or physical activity status (n = 3 males). While 18 children is a relatively small population, it represents 100% of the children recommended for the program.

### Procedures

The children were required to attend *FIT* over the course of 8 weeks on Monday, Tuesday, and Thursday evenings. The program was supported by registered dietitians, school physical education instructors, and health educators and volunteers from *HealthWorks!*, who were experienced in working with youth. The program’s physical activity and nutrition education curriculum was based on the American Council on Exercise’s *FitKids* program.^15^ Parents, and their children were required to attend the weekly nutrition education lessons, which were led each Monday night by a registered dietitian*.* The first hour of the nutrition education lesson was dedicated to classroom instruction on the topic of the week, and the second hour was dedicated to a question-and-answer session for parents and physical activity for the children. All physical activity was supervised by a licensed physical therapist and led by certified group fitness instructors.

Tuesday and Thursday evenings of the program were devoted to 1 hour of physical activity alone. Although all children received structured physical activity, the children were randomly assigned by drawing identification codes to a control or intervention group to determine if physical activities that reinforced the nutrition message of the week could help children better remember the nutrition education messages. Because the exercises were similar in intensity, the fitness measures were compared using pre- and post-intervention data for all of the children. The children in the control group participated in physical activities that were normally offered through the program, including calisthenics and group play. The children in the intervention group also took part in these activities, but each Thursday evening (6 times throughout the program) they participated in a nutrition-related physical activity during the last 10 minutes of regular exercise sessions. While not repeating the nutrition lesson, the activity was designed to reinforce the nutrition message that was delivered by the registered dietitian on Mondays. These lesson topics were developed from the selected consumer messages of *ChooseMyPlate.gov*^14^:

1. *ChooseMyPlate.* Students ran a relay race dropping food models into boxes labeled as the *MyPlate* food groups (protein, dairy, fruit, vegetables, and grains).
2. Label reading. Students ran intervals corresponding to nutrition labels on food models.
3. Serving size. As part of a game, students estimated serving sizes using food models.
4. Grocery store survival. Students participated in a scavenger hunt to find healthy food items in the *HeathWorks!* simulated grocery store.
5. Healthy snacking. Students ran a relay race and chose between healthy and not so healthy snacks with food models.
6. Healthy hydration. Students ran a relay race and chose between healthy and not so healthy drinks with drink models.

### Measures

A physical assessment of each child was conducted on the first night of the *FIT* program and the last night. This assessment included heights, weights, and blood pressure. Heights were measured using a portable wall-mounted measuring tape (White Stature Meter Height Measure Measuring Tape 200 cm/2M). Weights of the children, in light clothing and without shoes, were measured to the nearest 0.1 pound with a portable digital scale (Tanita HD-384 Digital Scale). Resting BP was measured using a CONTEC08A desktop electronic sphygmomanometer with a pediatric cuff (Contec Medical Systems Co., Ltd.; Qinhuangdao, Hebei Provice, People’s Republic of China). Measurements were taken with the subjects seated in an upright position, feet on the floor, and with the right arm at heart level.

Fitness parameters: numbers of push-ups, sit-ups, chair-squats, and step-ups were measured according to standard *Fitnessgram* protocol.^16^ Children were randomly divided into groups of 4 or 5 and rotated through each activity station with a 5 minute rest period between each station.

A 10-question nutrition quiz to assess nutrition knowledge was given on the first night and on last night of the program. Quizzes were adapted from validated pre- and post nutrition knowledge quizzes developed by Knight et al.^17^ The children completed the quizzes independently.

### Statistical analysis

Beginning and ending heights and weights and blood pressure measurements were analyzed using descriptive statistics. Pre- and post-intervention BMI was calculated for each student according to the following formula:

**Figure 1:**
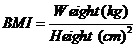

BMI was then converted to age and gender-specific z-scores according to the guidelines provided by the CDC^18^ and Knight et al.^19^ The use of BMI z-scores rather than direct BMI values helped to prevent factors such as differences in age and rate of growth from confounding the data. Pre- and post-intervention mean fitness test scores were compared using paired *t* tests with an alpha of 0.05. Because pre- and post-intervention mean height, BMI and BMI z-scores, scores were not normally distributed a Wilcoxon signed-rank test was used after observing the distribution of differences were symmetrically shaped.

Paired *t* tests were run on the treatment and control pre and post quiz scores. Two outliers were detected that were more than 1.5 box-lengths from the edge of the box in a boxplot however inspection of their values did not reveal them to be extreme and they were kept in the analysis. The difference scores for pre and post tests were normally distributed, as assessed by Shapiro-Wilk’s test (*P* = 0.181). The difference scores for the pre and post tests were normally distributed, as assessed by visual inspection of a normal Q-Q Plot.

Additionally, an analysis of covariance (ANCOVA) with a pretest-posttest design was used to test the for differences between intervention and control group quiz scores. There was a linear relationship between pre- and post-intervention quiz scores, as assessed by visual inspection of a scatterplot as well as homogeneity of regression slopes as the interaction term was not statistically significant, *F*_(1, 14)_ = 0.096, *P* = 0.762. Additionally, Standardized residuals for the interventions and the standardized residuals for the overall model were both normally distributed, as assessed by Shapiro-Wilk’s test (*P* > 0.05). There was homoscedasticity, as assessed by visual inspection of the standardized residuals plotted against the predicted values and there was homogeneity of variances, as assessed by Levene’s test of homogeneity of variance (*P* = 0.581). There were no outliers in the data, as assessed by no cases with standardized residuals greater than ±3 standard deviations. Chi-square analysis was used to determine differences in distribution of responses to individual questions from pre- to post-intervention survey questions. (IBM SPSS Statistics, Version 23 statistical software, 2015, Chicago, IL).

## Results

Anthropometric and blood pressure data is presented in Table 2. The females’ mean baseline weight of 57.89 (SD 18.01) was almost a full standard deviation from the males’ baseline weight of 50.96 (SD 15.31). When compared to the World Health Organization (WHO) health growth standards,^20^ the students’ mean BMI z-scores for females and males were indicative of health risk. The mean BMI z-score for girls, 2.046 (SD 0.543), was classified as “Overweight”, and the mean BMI z-score for males, 1.352 (SD 1.031), was in the “Possible risk of overweight” category. No significant changes in weight were seen at the end of the program.

When compared to blood pressure tables from the National Heart Lung and Blood Institute,^21^ the mean pre-*FIT* blood pressures for both females and males were around the 90th percentile. Significant (*P* < 0.05) differences between pre- and post-program means were seen for diastolic blood pressure in girls, changing from 67.82 (SD 6.00) to 59.90 (SD 9.31) mm Hg and for the group as a whole [66.63 (SD 8.81) to 63.75 (SD 11.81) mm Hg)].

The means for the fitness test results are found in Table 3 and when compared to the *Fitnessgram* performance standards,^16^ indicate that the students in this study were already relatively fit. The mean fitness scores from both pre- and post-*FIT* exceeded the *Fitnessgram* performance standards in every category. Significant (*P* < 0.05) differences were seen from pre-program to post-program for the girls in push-ups [14.00 (SD 6.23) to 20.2 (SD 5.63)] and chair squats [29.3 (SD 3.23) to 32.4 (SD 4.59)] which in turned affected significant differences for the total group for push-ups [14.31 (SD 7.62) to 19.63 (SD 6.62)] and chair squats [30.50 (SD 10.21) to 34.44 (SD 7.39)]. Significant (*P* < 0.05) differences were seen from pre-program to post-program for sit-ups for the total group [39.63 (SD 5.80) to 44.38 (SD 4.91)].

Paired between *t* test for both control and intervention groups indicated that test scores in the treatment group increased significantly 1.667 (95% CI, 0.392 to 2.941) points between pre and post testing (t _df 8_ = 3.015, *P* = 0.017), while the control group did not result in a statistically significantly increase between pre and post testing 1.000 (95% CI, -0.215 to 2.215) points t _df 8_ = 1.897, *P* = 0.094. Analysis of covariance indicated that when taken as a whole test scores, reported here as mean ± standard deviation, of the intervention group were higher (8.33 ± 1.60) compared to those of the control group (6.33 ± 1.11). Adjusting for pre-intervention test scores, there was a statistically significant difference in with and without intervention scores, F (*df*=1, 15) = 6.367, *P* = 0.023.

Chi-square analysis of the mean student scores for the pre- and post-*FIT* nutrition and fitness quiz between students who received the nutrition education reinforcing physical activity and the control group showed no significant difference between the pre- and post-intervention mean quiz scores. However, both groups, answered more questions correctly on the post-intervention quizzes compared to the pre-intervention quizzes. The control group answered 12 more questions correctly on the post-intervention quiz compared to the pre-intervention quiz while the treatment group had 17 more correct answers. The control group also had 4 incorrect answers when comparing pre- to post-intervention quizzes while the intervention group had only 2 incorrect answers. Students who received the intervention answered correctly more often that the control group to questions about amount of servings of fruit per day, how much of the plate should be fruits and vegetables, calorie level of foods labeled “low fat”, and what is a balanced exercise plan. All student participants correctly identified the importance of warm up before exercise. Even on questions where the intervention group did not perform significantly better than the control group, the intervention group still scored higher.

## Discussion

Weight did not go down for the total group or for either gender, but this may have been due to the large standard deviation in weights of growing children. Similar studies have reported that short-term programs do not typically see a decrease in BMI, especially in children and should not be the sole indicator of a weight management programs success.^10^

Other health parameters, namely diastolic blood pressure in girls and resting heart rate for boys, were improved. The American Heart Association encourages children to maintain a healthy weight, participate in regular physical activity, and consume a heart-healthy diet in order to lower risk of high blood pressure which is a risk factor for heart disease.^22^ The lowering of diastolic blood pressure may have been due to the increased fitness levels that were also seen in the children, as the number of push-ups, chair squats, and sit-ups that the children were able to do increased for the whole group. In a systematic review of the health benefits of physical activity in school-aged children, Janssen and LeBlanc^23^ documented studies that compared kind and amounts of physical activity with various health parameters. They concluded that even modest amounts of physical activity can have health benefits in obese children. Although the relationship between blood pressure and fitness was the weakest of all the parameters, reduction in systolic and diastolic blood pressure were seen when children participated in aerobic physical activity.^24,25^

All the children performed better on the post-*FIT* Nutrition Quiz than the pre-*FIT* Nutrition Quiz. However, the survey/nutrition quiz results comparing control and treatment post-test scores showed that there was a positive change in nutrition knowledge with reinforcing activity: on the post-FIT Nutrition Quiz; the treatment group did the same or better on every question than the control group and scored higher on the quizzes overall. In a study to increase knowledge retention of nursing students in a cardiology lecture, Wagner^26^ reported that a concept-based, student-centered approach using active and kinesthetic learning activities can enhance engagement and improve clinical problem solving, communication skills, and critical thinking. Brull and Finlayson^27^ suggested that many learners would benefit from “gamification”, the application of game features such as elements, mechanics, aesthetics, and metaphors into non-game settings like education. Faiella and Ricciardi^28^ stated that the literature on gamification affirms that the thoughtful use of game elements can produce a learning situation characterized by high motivation and engagement. The reinforcing activities did seem to help the students learn in this intervention. Further research should be conducted to determine the effect of knowledge-reinforcing physical activity on long-term knowledge retention.

Overall, physical activity was effective in a short-term nutrition education and physical activity program for children and their families and did seem to reinforce the nutrition education offered in this short-term program. Gately et al^29^ determined that a short-term residential weight-loss program was effective across a range of health outcomes and this study supports that conclusion. Although, BMI z-scores did not change, the decreases in diastolic blood pressure for girls and resting heart rate for boys and increase in fitness parameters indicate that short-term weight management programs can produce positive health effects.

## Ethical approval

The Institutional Review Boards of both the University of Mississippi and North Mississippi Health Services approved the *FIT* program and this evaluation study.

## Competing interests

We declare no conflicts of interest.

## Authors’ contributions

KBK, SAD, MM, and KT conceptualized the study, designed data collection methodology, analyzed the data, and led manuscript development. SAD, MM, and HW collected data. SSK analyzed the data. AKB and SSK assisted with manuscript development.

## Acknowledgments

No funding was used to prepare this manuscript. The *FIT* program was funded by North Mississippi Health Services.

**Table 1:** Demographic characteristics of *FIT* participants

**Variable**	**Students (n = 18)**	**Parents (n=13)**
Mean age (y)	10.52 (1.26)	42.5 (3.92)
Gender		
Males (n=9)	50%	8%
Females (n=9)	50%	92%
Ethnicity		
White	56%	62%
Black	25%	23%
Hispanic	0%	0%
Asian	0%	8%
Multiracial	19%	0%
Other	0%	8%
Residence		
Apartment	13%	8%
House	75%	77%
Mobile home	13%	15%
Education level (parents only)		
8^th^ grade		0%
High School/GED		0%
College/University		85%
Graduate/Professional		15%

Standard deviations are reported in parentheses.

**Table 2:** Anthropometric and blood pressure data for *FIT* student participants

**Measure**	**Females**	**Males**	**Total**
Height (cm)			
Pre	142.38 (11.19)	140.61 (16.18)	142.57 (12.42)
Post	143.58 (11.56)	144.62 (13.29)	143.71 (12.32)
Weight (kg)			
Pre	57.89 (18.01)	50.96 (15.31)	52.51 (16.42)
Post	60.17 (18.66)	49.96 (15.54)	53.52 (16.76)
BMI (kg/m^2^)			
Pre	27.17 (7.65)	23.53 (5.25)	25.49 (5.87)
Post	28.70 (6.36)	23.69 (5.74)	25.56 (5.74)
BMI z score			
Pre	2.046 (0.543)	1.352 (1.031)	1.72 (0.86)
Post	2.161 (0.427)	1.340 (0.990)	1.78 (0.84)
Systolic BP (mm Hg)			
Pre	113 (12.24)	110.63 (12.83)	111.31 (12.42)
Post	113 (11.69)	109.00 (12.90)	112.25 (13.55)
Diastolic BP (mm Hg)			
Pre	67.82 (6.00)*	60.00 (10.86)	66.63 (8.81)*
Post	59.90 (9.31)*	66.00 (12.82)	63.75 (11.81)*

Standard deviations are reported in parentheses. * P < 0.05.

**Table 3:** Mean (SD) pre- and post-*FIT* student fitness test results

**Fitness parameters**	**Females (n=9)**		**Males (n=11)**		**Total (n=20)**	
	**Pre-** ***FIT***	**Post-** ***FIT***	**Pre-** ***FIT ***	**Post-** ***FIT***	**Pre-** ***FIT ***	**Post-** ***FIT***
Push-ups	14.00 (6.23)	20.21 (5.63)*	12.11 (7.37)	15.52 (9.84)	14.31 (7.62)	19.63 (6.62)*
Sit-ups	37.82 (6.72)	42.91 (6.66)	39.70 (6.08)	4.75 (4.98)	39.63 ± 5.80	44.38 (4.91)*
Chair squats	29.30 (3.23)	32.48 (4.59)*	34.78 (13.27)	36.38 (8.81)	30.50 ± 10.21	34.44 (7.39)*
Step test	122.61 (14.25)	122.92 7.05)	121.78 (14.01)	162.89 (6.55)*	123.06 (12.27)	149.13 11.19)*

Standard deviations are reported in parentheses. * *P* ≤ 0.05.
